# The Inhibitor of Apoptosis (IAPs) in Adaptive Response to Cellular Stress

**DOI:** 10.3390/cells1040711

**Published:** 2012-10-10

**Authors:** Arthur Marivin, Jean Berthelet, Stéphanie Plenchette, Laurence Dubrez

**Affiliations:** 1 Institut National de la Santé et de la Recherche Médicale (Inserm), UMR866, Dijon F-21079, France; Email: arthurmarivin@gmail.com (A.M.); berthelet.jean@gmail.com (J.B.); 2 Institut Fédératif de Recherche (IFR), Université de Bourgogne, 100, Dijon F-21079, France; Email: stephanie.plenchette@u-bourgogne.fr; 3 Ecole Pratique des Hautes Etudes (EPHE) EA 7269, Dijon, F-21079, France

**Keywords:** IAPs, apoptosis, caspases, NF-κB, TNFR, UPR, DNA damage response, cancer, neurodegenerative disease

## Abstract

Cells are constantly exposed to endogenous and exogenous cellular injuries. They cope with stressful stimuli by adapting their metabolism and activating various “guardian molecules.” These pro-survival factors protect essential cell constituents, prevent cell death, and possibly repair cellular damages. The Inhibitor of Apoptosis (IAPs) proteins display both anti-apoptotic and pro-survival properties and their expression can be induced by a variety of cellular stress such as hypoxia, endoplasmic reticular stress and DNA damage. Thus, IAPs can confer tolerance to cellular stress. This review presents the anti-apoptotic and survival functions of IAPs and their role in the adaptive response to cellular stress. The involvement of IAPs in human physiology and diseases in connection with a breakdown of cellular homeostasis will be discussed.

## 1. Introduction

Cells are constantly exposed to various endogenous and exogenous stressful conditions which can inflict serious damage. The ability of a cell to detect and adapt to environmental changes is essential to preserve cellular and tissue function during development and adult life. Some cells are more exposed to environmental aggression and require important protective mechanisms, such as: neuronal cells with low regenerative powers, cells from the innate immune response that are exposed to pathogens and pro-inflammatory environment, and hepatocytes exposed to xenobiotics. Cells have developed protective and adaptive mechanisms which include a slowing down of cellular metabolism, up-regulation of sensor molecules that detect damages, the expression of chaperones that protect essential cell constituents, and an increase and stabilization of pro-survival molecules. In cases of irreversible damage, tissue integrity is preserved by the activation of programmed cell death. Deregulation of these processes can lead to serious disorders. For example, a sustained expression of pro-survival molecules may lead to the spread and accumulation of cellular damages responsible for promoting tumors. Conversely, an exacerbated cell death can lead to tissue degradation that characterizes autoimmune and neurodegenerative diseases.

IAPs (Inhibitor of Apoptosis) are a family of proteins that regulate cell death as well as survival pathways. They determine cell fate in response to a stimulation of the death receptor superfamily, cytokine and antigen receptors, membrane and cytoplasmic sensors of microbiological patterns, and also to DNA damaging agent exposure, favoring cell survival [1,2]. They are potent regulators of innate immune response [2,3]. The expression of some IAPs can be rapidly induced under stressful conditions due to the presence of an internal ribosome entry site (IRES)-dependent mechanism of translation initiation [4,5,6]. This article will present the essential properties of the IAPs and discuss their contribution to the adaptive response to cellular stress. Their involvement in the innate immunity has been extensively reviewed elsewhere [2,3] and will not be developed here.

## 2. The Inhibitor of Apoptosis (IAP) Family of Proteins

IAPs (also named BIRC for BIR (baculoviral IAP repeat) containing proteins) belong to an evolutionary conserved family of proteins, first identified in baculovirus for their anti-apoptotic properties. The IAP family contains eight mammalian members: neuronal apoptosis inhibitory protein (NAIP, also named BIRC1), cellular IAP1 (cIAP1 or BIRC2), cellular IAP2 (cIAP2 or BIRC3), X chromosome-linked IAP (XIAP or BIRC4), survivin (also named BIRC5), BIR repeat-containing ubiquitin-conjugating (Bruce)/Apollon (or BIRC6), melanoma IAP (ML-IAP or BIRC7), and IAP-like protein 2 (ILP2 or BIRC8) [1,2].

### 2.1. Structural Feature and Molecular Functions

IAP family members harbor a combination of several conserved protein domains that determine their molecular functions [1]. They are defined by the presence of at least one BIR domain essential for their interaction with protein partners and for their anti-apoptotic activity [7]. In addition to the BIRs, most IAPs contain a domain involved in the ubiquitination process such as a RING (really interesting new gene) or UBC (Ub-conjugating) domain [8]. This post-translational modification consists in the covalent binding to a lysine (K) of target proteins of an ubiquitin (Ub) molecule or Ub chains. Ub chains are formed by conjugation of the C-terminal Glycine (G76) of Ub to a K residue (mainly K11, K48, K63) of another. Ub can also be attached to the N-terminal residue, forming a linear Ub chain. Ubiquitination is a multi-steps process involving the successive action of Ub activating enzymes (E1), Ub-conjugating enzymes (E2) and Ub protein ligases (E3) that confer substrate specificity [8]. cIAP1, cIAP2, XIAP, ML-IAP, and ILP2 are E3-Ub ligases thanks to the presence of a C-terminal RING domain. This domain catalyzes the transfer of mono-Ub or poly-Ub chains to protein substrates. Furthermore, the RING enables homo- or heterodimerization of cIAP1, cIAP2 and XIAP that regulates their stability and possibly their activity [9,10,11,12,13,14]. The giant IAP Apollon is an E2-conjugating enzyme thanks to the presence of a C-terminal UBC domain [15]. cIAP1, cIAP2 and XIAP also contain a UBA (Ub associated) domain that can recognize mono- and poly-Ub chains and that allows the recruitment of cIAP1 in protein complexes [16,17]. Moreover, cIAP1 and cIAP2 own a central CARD (caspase recruitment domain) with regulatory functions [18]. Lastly, NAIP contains NACHT (domain present in NAIP, CIITA, HIT E and TP1) and LRR (leucine-rich repeat) domains which characterize NLR (NOD (nucleotide binding and oligomerization domain)-like receptors) [19].

### 2.2. IAPs as Inhibitor of Cell Death

Overexpression of IAPs can protect cells against apoptotic stimuli. Moreover, deletion or inhibition of IAPs can sensitize cancer cells to chemotherapy, radiotherapy or cell death agonistic treatment [20]. The anti-apoptotic activities of IAPs were initially attributed to their capacity to bind some caspases (cysteine-dependent aspartate-specific protease), central effectors of apoptosis. However, recent studies have shown that IAPs can also inhibit cell death at an early stage, preventing the formation of multi-protein platforms that can induce caspase-dependent and independent cell death. 

#### 2.2.1. Apoptotic Signaling Pathways

**Figure 1:**
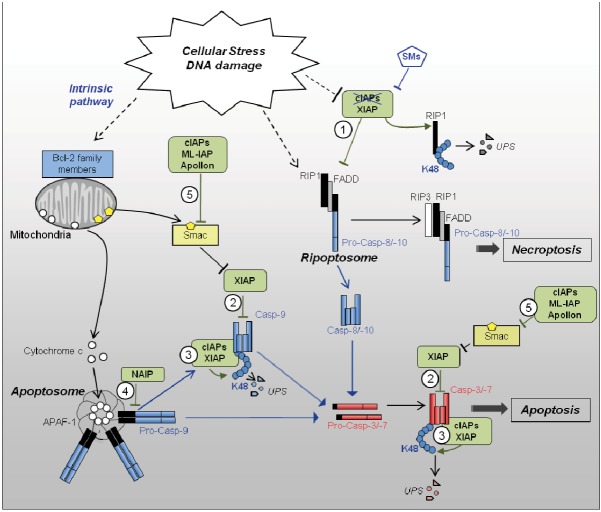
Regulation of cellular stress-induced cell death pathways by IAPs. Cellular stress likely activates the intrinsic pathway of cell death involving the release from the mitochondria, of pro-apoptotic factors including cytochrome c and Smac. Once in the cytoplasm, cytochrome c induces an ATP-dependent conformational change and oligomerization of APAF-1 (apoptotic peptidase activating factor-1) in the Apoptosome. APAF-1 then recruits and promotes the activation, through homodimerization, of the initiator caspase-9. Active caspase-9 is then stabilized by auto-processing and detached from apoptosome. DNA damage can also induce a depletion of cIAP1 and XIAP, allowing the formation of a RIP-1 containing platform named Ripoptosome. Ripoptosome can trigger either caspase-8 or -10 activation and apoptosis, or caspase-independent cell death referred to as Necroptosis. Initiator caspases-9, -8 or -10 induce the activating proteolytic processing of effector caspase-3 and/or -7 responsible for apoptotic cell death. IAPs can inhibit apoptotic pathways at several levels: 1) cIAPs and XIAP are potent inhibitors of Ripoptosome assembly; 2) XIAP can directly inhibit the activity of processed forms of caspase-9, -3 or -7; cIAPs;3) XIAP can induce the K48 ubiquitination and proteasome-mediated degradation of processed forms of caspase-9, -3 or -7; 4) NAIP is able to block the proteolytic processing of caspase-9 at the Apoptosome level; 5) cIAPs, ML-IAP and Apollon can bind to Smac, preventing it from neutralizing XIAP. SMs: Smac mimetics; UPS: ubiquitin-proteasome system.

Apoptosis is a multi-step proteolytic process involving a family of cysteine proteases named caspases. They are synthetized as a single chain zymogen consisting of one pro-domain and two active sub-units (a small and a large one). The apoptotic caspases are subdivided into two subgroups: the initiators caspases (caspase-2, -8, -9 and -10) which activate the effectors (caspase-3 and -7) responsible for cell dismantlement. The initiator caspases are activated by homo-dimerization through their recruitment by an adaptor protein, in a multi-protein activation platform. Once activated, they undergo an autocatalytic cleavage that releases the two active sub-units, which assemble into a heterotetramer composed of two small and two large subunits. Active effector caspases are heterotetramers formed after proteolytic processing mediated by initiator caspases [21] (Figure 1).

**Figure 2:**
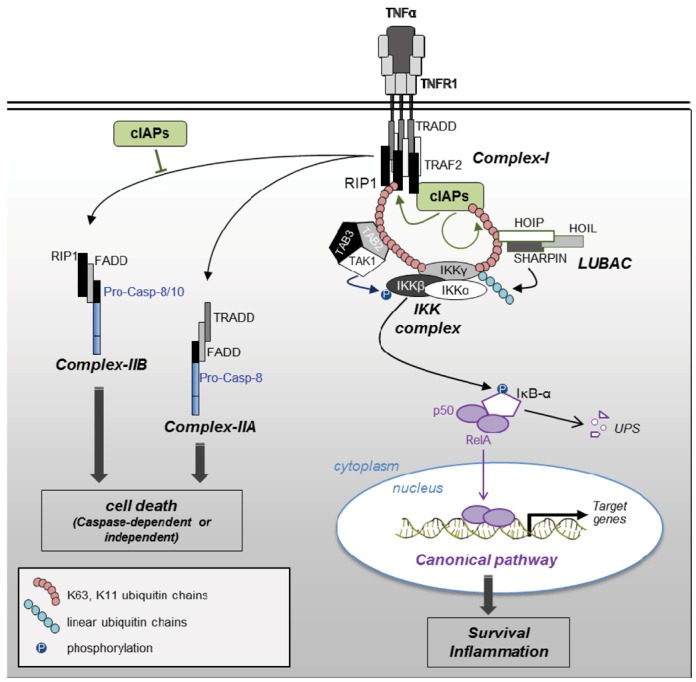
Regulation of TNFRI-signaling pathways by IAPs.TNF-R1 stimulation induces the recruitment to the receptor, of cIAPs and RIP1 via TRADD and TRAF2 into complex-I. cIAPs trigger K63 self-ubiquitination and K11 and K63 RIP1 polyubiquitination. Ub chains are recognized by Ub-binding domain of TAB2, IKKγ and HOIP and connect TAB2/TAB3/TAK1, IKK complex and LUBAC, resulting to LUBAC-mediated linear ubiquitination of IKKγ and TAK1-mediated phosphorylation of IKKβ. Once activated, IKK complex triggers phosphorylation of IκB-α, that is then degraded by UPS (ubiquitin proteasome system). Released NF-κB dimer translocates to the nucleus and promotes the transcription of target genes. Secondary cytoplasmic complex leading to cell death can be formed in absence of IAPs (complex-IIB) or when NF-κB signaling is defective (complex-IIA).

Several initiator caspase-activating platforms were described depending on apoptotic stimuli (reviewed by Mace & Reidl [22]):

(1) Apoptosome is the caspase-9-activating platform involved in the mitochondria-mediated pathway (intrinsic pathway) of apoptosis. It is activated by intracellular stress, or nutriment deprivations which induce Bcl-2 (B-cell lymphoma-2) family members’ controlled-mitochondrial outer membrane permeabilization, resulting in the release of pro-apoptotic molecules including cytochrome c and Smac/DIABLO (second mitochondria-derived activator of caspases/direct IAP-binding protein with low pI) (Figure 1). Cytoplasmic cytochrome c triggers ATP-dependent conformational change and oligomerization of the adaptor APAF-1 (Apoptotic peptidase activating factor 1) in a high-molecular mass cytoplasmic complex referred to as Apoptosome. APAF-1 then recruits pro-caspase-9 leading to its activation. Once activated, caspase-9 is auto-processed, and then quickly disconnected from the apoptosome and inactivated [23]. 

(2) DISC (death-inducing signaling complex) is a receptor associated protein complex formed by the stimulation of death domain containing cell surface receptors from a tumor necrosis factor receptor (TNFR) superfamily such as CD95 (Fas, DR2), and the Trail receptors DR4 and DR5. Binding of ligand to trimeric receptor induces the recruitment of the adaptor FADD (Fas-associated death domain protein) and pro-caspase-8 or -10, leading to caspase activation [22].

(3) Tumor necrosis factor receptor 1 (TNFR1) engagement triggers the assembly of a membrane-localized multi-protein complex called complex-I which includes TRADD (TNFR1-associated Death domain), TRAF2 (TNFR-associated factor 2), cIAPs, and the kinase RIP1 (Receptor interacting protein kinase 1). Complex-I initiates an ubiquitin-dependent transduction of survival or pro-inflammatory signals. When NF-κB signaling is blocked or in the absence of cIAPs, secondary cytoplasmic caspase-8-activating complexes can be formed from the first one. These cell-death inducing complexes are composed of TRADD, FADD and caspase-8 (referred to as Complex-IIA) when NF-κB signaling is defective and of RIP1, FADD and caspase-8 (referred to as Complex-IIB) in the absence of cIAPs [22] (Figure 2).

(4) Recently, a new cytoplasmic caspase-8-activating platform named Ripoptosome has been described [24,25] (for review, see [26]). The core components of Ripoptosome are RIP1, FADD and caspase-8. The Ripoptosome formation requires the kinase-activity of RIP1. It is assembled independently of death receptor and mitochondrial pathways, in response to genotoxic stress or TLR3 stimulation (Toll-like receptor 3) [24,25] and can trigger both caspase-dependent and independent cell death (Figure 1).

(5) Genotoxic stress can also induce oligomerization of RAIDD (receptor-interacting protein-associated ICH-1/CED-3 homologous protein with a death domain) and PIDD (p53-induced protein with death domain) in a soluble platform named PIDDosome. RAIDD then recruits and activates caspase-2 [22]. Caspase-2 can also be activated in response of ER stress or bacterial toxin in a high-molecular-weight complex independent from PIDDosome, but the exact nature of this complex is not well established [1,27,28,29].

#### 2.2.2. Regulation of Caspases by IAPs (Figure 1)

XIAP, cIAPs and NAIP can directly bind some caspases with varying consequences depending on the IAP and caspase concerned, and possibly the cellular context. BIR2 and BIR3 domains of these IAPs contain a surface groove able to bind a short peptide named IBM (IAP binding motif) found in the N-terminus of active sub-units of caspase-3, -7, -9 [30]. BIRs can bind to the IBM peptide when exposed to the N-terminal extremity of the protein. Thus, the BIR IBM groove can only bind processed, activated caspases. All BIRs display differential binding specificity, depending on the amino-acid composition of the core recognition motif of IBM groove [7]. An IBM is also found in the N-terminus of Smac/Diablo released from the mitochondria in response to apoptotic stimuli, and which acts as a competitive inhibitor of the IBM-dependent interaction of IAPs with caspases [15,31,32,33,34]. The Smac IBM was used to design IAP antagonists named Smac mimetics (SMs), currently under clinical investigation [20].

In contrast to the initial statement, XIAP is the only IAP able to directly inhibit enzymatic activity of caspases [35]. It binds to processed caspases-3, 7 and -9 by a two-site binding mechanism [36,37,38]. First, XIAP docks active caspase-3, or -9 via its BIR2 or BIR3 IBM-binding groove, respectively. Second, the BIR3 binds the dimer interface of caspase-9, or the linker region upstream of BIR2 binds across the substrate binding pocket of caspase-3 and -7, which hinder substrate accessibility and hide the catalytic residue [36,37,38]. Moreover, XIAP is able to induce the K48 ubiquitination of active caspase-3 leading to its degradation [39,40], and the neddylation of caspase-7 inhibiting its activity [41]. The analysis of apoptosis in fibroblasts and thymocytes from XIAP∆RING transgenic mice revealed the importance of the RING-dependent post-translational modifications for the inhibition of caspases [42]. Interestingly, the capacity of XIAP to control capase-9 activity appears to be directly correlated with the level of APAF-1 and apoptosome activity [43]. Thus, XIAP effectively regulates sensitivity to apoptotic stimuli in cells expressing a low level of APAF-1 such as terminally differentiated neuronal cells and cardiomyocytes [44,45]. 

cIAPs can interact with caspase-9 and caspase-7 in an IBM-dependent manner, and with the pro-domain of intermediary processed form of caspase-3 (p20_2_/p12_2_), independently of IBM [30,46]. Although unable to inhibit enzymatic activity of caspases, cIAPs can regulate the stability of active tetrameric caspases through a UPS (Ub Proteasome system)-dependent mechanism [46]. Moreover, cIAP2 is able to induce a non degradative mono-ubiquitination of caspase-3 and -7 [47], suggesting that mammalian IAPs could also regulate caspases through UPS-independent, Ub-dependent mechanisms, as observed in Drosophila [48]. In addition, cIAPs, as well as ML-IAP and Apollon, can bind to SMAC, preventing it from neutralizing XIAP [15,31,32].

In contrast to other IAPs, NAIP can interact with pro-caspase-9 when present in the apoptosome complex, and inhibits pro-caspase-9 cleavage. This interaction involves the BIR3 domain of NAIP and is IBM-independent [49]. Thus, NAIP can inhibit the intrinsic pathway of apoptosis at an early step (Figure 1).

#### 2.2.3. Blockade of RIP1-Containing Cell Death-Inducing Platform Formation

The influence of IAPs on cell death-inducing platform formation was first highlighted by the use of SMs which induce a very rapid degradation of cIAPs [16,24,50,51,52]. These molecules considerably sensitize cells to cell death induced by TNFα exposure [52,53,54,55,56,57]. In the absence of cIAPs, TNFR1 stimulation rapidly induces the assembly of caspase-8-activating complex-II leading to cell death. In some tumor cells which are sensitive to SMs, cIAPs depletion induces a spontaneous formation of Ripoptosome [24,25] or leads to an NF-κB (nuclear factor-kappa B)-dependent production of TNFα, which triggers cell death via an autocrine pathway [50,52,55,56,57,58].

cIAPs are potent regulators of RIP kinases [24,53,54,58,59,60]. They can mediate the conjugation of K11, K48 and K63 and linear Ub chains [53,58,59,60,61,62]. Upon TNFR1 stimulation (Figure 2), RIP1 is recruited to cell surface receptor complex–I and subjected to cIAP1-mediated K11 and K63 poly-ubiquitination [53,54,62]. These Ub chains serve as a signal for the activation of the NF-κB signaling pathway as explained below (paragraph 2.3.1). In the absence of cIAPs, RIP1 is recruited to a secondary cytoplasmic platform (complex-IIB) containing RIP1, FADD and caspase-8 or -10 and leading to cell death (Figure 2). Thus, cIAPs are important determinants of the TNFα response, promoting pro-survival or pro-inflammatory signals and blocking the cell death signal. A cIAP-regulated-assembly of RIP1 containing cytoplasmic cell death complexes can also be triggered by TRAILR, or CD95 stimulation [22,60] or independently of the death receptor (referred to as Ripoptosome) after TLR3 stimulation, Tweak engagement or genotoxic stress [24,25,26]. Several mechanisms of regulation of RIP1-containing platforms assembly by cIAPs have been proposed, including K48 ubiquitination leading to degradation of Ripoptosome constituents, or non-degradative ubiquitination of RIP1 which can block RIP1 kinase activity and/or cell death complex assembly [25,58]. RIP1-containing platforms can elicit apoptotic response as well as caspase-independent cell death referred to as necroptosis (programmed form of necrosis). The decision between the two cell death pathways depends on the presence of RIP3 that is indispensable for necroptosis, the long or short isoforms of cellular FLICE-inhibitory protein (cFLIP), and the generation of reactive oxygen species [63]. Besides RIP1, cIAPs can also ubiquitinate other members of the RIP kinase family including RIP3 [61], however, the consequence of cIAPs-mediated RIP3 ubiquitination was not established. The capacity of XIAP to negatively regulate Ripoptosome formation has also been observed. XIAP is also able to bind and ubiquitinate RIP1 [25]. The understanding of the mechanisms of regulation of RIP-containing platform assembly and activity and the individual contribution of IAPs are still subjected to debate and will require more investigations. Since IAPs can form homo- and heterodimer [11,12,13], it is possible that IAPs cooperate to regulate the RIP-containing complex. The recent analysis of cIAP1/XIAP and cIAP1/cIAP2 double knockout mice demonstrated that deletion of RIP1 or RIP3 rescues cIAP1/XIAP double knockout mice from embryonic death and prolonged cIAP1/cIAP2 double knockout mice embryonic survival [64] highlighting the importance of IAPs in the control of RIP-containing cell death platform in development.

### 2.3. Pro-Survival Properties of IAPs

#### 2.3.1. NF-κB Activating Signaling Pathways

The role of IAPs in innate immunity, through regulation of the NF-κB activating signaling pathways is well documented (for reviews, see [2,3]). NF-κB correspond to a family of transcription factors induced by the stimulation of cytokines (TNFα or IL-1), antigen receptors, or recognition of microbiological patterns by TLR, NOD (Nucleotide-binding oligomerization domain-containing protein) or NLR (NOD-like receptor) family of proteins. They are required for the expression of pro-inflammatory molecules in response to microbial aggression. NF-κB are also important regulators of gene transcription involved in cell survival, differentiation and proliferation. They are activated in response to DNA damage and reactive oxygen species and take part of the adaptive response [65]. 

The active form of the transcription factor is a dimer formed by one Rel sub-unit (Rel A also called p65, RelB or c-Rel) and one NF-κB sub-unit (p50 or NF-κB1 and p52 of NF-κB2). Two main NF-κB activating pathways were described: Canonical or classical, and noncanonical or alternative. They are both regulated by phosphorylation and ubiquitination processes (for review, see [65]). IAPs appear to regulate both, promoting the canonical and blocking the noncanonical pathway.

Canonical NF-κB pathway. It mainly concerns the p50/RelA dimer sequestered in its inactive form in the cytoplasm by IκB (Inibitor κB) proteins. Upon stimulation of cell surface or intracellular receptors, or DNA damage, IκB-α is degraded by UPS after phosphorylation by IKKβ (IκB kinase β), associated with the kinase IKKα, and the regulatory protein IKKγ (also named NEMO for NF-κB essential modulator) in the IKK complex. Released NF-κB dimer translocates to the nucleus and promotes the transcription of target genes. IKK complex activity requires ubiquitination of IKKγ and the phosphorylation of IKKβ by the kinase TAK1 (TGFβ-activated protein kinase 1-binding protein) [65,66,67]. 

cIAPs have been involved in cell surface receptors-mediated NF-κB activation by promoting stearic proximity of the IKK complex, LUBAC and TAK1/TAB2/TAB3 (TGFβ-activated protein kinase 1-binding protein) [68] (Figure 2). Upon TNF-R1 stimulation, both cIAPs and RIP1 were recruited to TNF-receptor complex via TRADD and TRAF2 [22,69]. cIAPs trigger K63 self-ubiquitination and K11 and K63 RIP1 polyubiquitination [53,54,58,62]. These Ub chains are a signal recognized by Ub-binding domain (UBD) of TAB2, IKKγ and the LUBAC component HOIP (HOIL (Heme-oxidized IRP2 Ub ligase-1) interacting protein) [58,62,69]. A regulation of NF-κB activating the signaling pathway by XIAP has also been demonstrated through its capacity to activate TAK1 and connect TAK1/TAB2/TAB3 to IKK complexes [66,70].

Moreover, cIAP1 can catalyze the monoubiquitination of IKKγ on K277, K309 and K285 [66,71]. IKKγ monoubiquination has been proposed as favoring its nuclear export. K285 monoubiquitination is essential for IKKγ activation by genotoxic stress [71]. The mechanisms are not clearly identified. Since K285 is also an acceptor site of linear ubiquitination required for genotoxic-induced NF-κB activation [67], we may suppose that cIAP1 could cooperate with LUBAC in this process.

Noncanonical NF-kB pathway. The alternative pathway triggers the activation of p52 NF-κB2 from its p100 precursor via limited proteasomal-mediated proteolysis. In contrast to the canonical pathway, the noncanonical pathway is independent of IKKγ. It involves the kinase NIK (NF-κB-inducing kinase) that catalyzes the phosphorylation of IKKα, that in turn induces the phosphorylation of p100 required for its activation. In resting cells, cIAPs catalyze K48 ubiquitination of NIK that targets the protein for proteasomal-mediated degradation. The recruitment of cIAPs along with their partners TRAF2 and TRAF3, to CD30, CD40 or the TWEAK/FN14 receptor upon ligand stimulation, or deletion of cIAPs by SMs releases NIK, which activates NF-κB noncanonical pathway [72,73]. 

#### 2.3.2. Cell Proliferation

Besides Survivin, the atypical IAP that regulates chromosome alignment and segregation during mitosis and cytokinesis [74], cIAPs are able to regulate cell proliferation. cIAP1 is almost exclusively localized in the nuclei of self-renewing hematopoietic stem cells, and is excluded from the nuclei during differentiation [75,76,77]. A nuclear localization of cIAP1 has also been observed in colic, mammary and skin epithelial cells and in some tumor cell lines [75]. In these cells, the silencing of cIAP1 decreases cell proliferation that is associated with a significant increase in G0/G1 phases of cell cycle and a down-regulation of Cyclin E and A. The research of IAP partners in the nucleus compartment revealed an interaction of cIAP1 with the transcription factor E2F1. cIAP1 is recruited along with the transcription factor to DNA promoter and favors its transcriptional activity. It appears to be required for the recruitment of E2F1 to DNA [75]. cIAP1 can also control cell proliferation and differentiation through regulation of c-myc. c-myc is a large spectra transcription factor controlling cell survival, proliferation and differentiation [78]. It is a very early response gene to cellular stress and its deregulation contributes to the development of a wide variety of human cancers. cIAP1 indirectly stimulates c-myc by inducing the ubiquitination and proteasomal-mediated degradation of its antagonist Mad-1 (Max-dimerization protein-1) [79].

## 3. Role of IAPs in Adaptive Response to Cellular Stress (Summarized in Table 1)

Cells subjected to transient stressful conditions ensure survival by freezing their intra-cellular metabolism and by setting up protective mechanisms. Thus, cells rapidly induce the expression of specific molecular chaperones that protect proteins from damages, and pro-survival molecules that counteract cell death response. Among these proteins, cIAP1 and XIAP are rapidly upregulated due to the presence of a cap-independent mechanism of translational initiation. Generally, translation is initiated by the recruitment of initiation factors and ribosomal sub-unit to the 5' end cap structure of the mRNA. The translation of cIAP1 and XIAP is regulated by an IRES sequence element located in the 5' untranslated region (UTR) of the mRNA [4,5,6,80,81]. The IRES can rapidly recruit ribosome sub-unit, and is preferentially used under stressful conditions when the cap-dependent mechanism is reduced. Thus hypoxia, serum starvation, low-dose gamma irradiation and ER (Endoplasmic Reticulum) stress induced a very quick translational up-regulation of XIAP and/or cIAP1 [4,5,6,80,82]. Moreover, the stability of IAPs are regulated by HSPs (heat shock proteins) suggesting an essential function of IAPs in adaptive response to cellular stress [77].

**Table 1:** Influence of IAPs in adaptive response to cellular stress.

Cellular Stress	IAP	Regulation (-) and effects (→)		Ref.
UPR	cIAP1	-	IRES-dependent translational up-regulation	[4]
		-	PERK-dependent transcriptional and translational up-regulation	[84]
		-	P3K dependent transcriptional up-regulation	[85]
			➨ Cell death protection	[84,85]
	cIAP2	-	PERK-dependent transcriptional and translational up-regulation	[84]
			➨ Cell death protection	
	XIAP	-	P3K dependent transcriptional up-regulation	[85]
			➨ Cell death protection	
DNA damage	cIAP1	-	IRES dependent translational up-regulation	[103]
			➨ Cell death protection	
			➨ Canonical NF-κB activation through ubiquitination of IKKγ	[71]
		-	Auto-ubiquination and degradation	[14,25]
			➨ Ripoptosome formation and cell death	[25]
	XIAP	-	MDM2 and IRES dependent up-regulation	[80,81]
			➨ Cell death protection	[81]
			➨ Canonical NF-κB activation through bridging of IKK complex and TAK1	[66]
		-	Auto-ubiquination and degradation	[14,25]
			➨ Ripoptosome formation and cell death	
Pro-inflammatory environment	cIAP2	-	Up-regulation upon activation by LPS	[109,110,111]
			➨ Macrophages survival in pro-inflammatory environment	[109]
	cIAPs		➨ Protective effect	[113]
			➨ Cell fate decision in response to TNFα or pathogen recognition	Reviewed in [2,3]
Hypoxia and ischemia	XIAP		➨ Neuro-protective effect	[88,89]
	NAIP		➨ Neuro-protective effect	[101,102]
Oxidative stress	XIAP		➨ Regulates the level of expression of anti-oxidant enzymes →reduced intracellular ROS	[94,95]
			➨ Ubiquitination of copper transporters →regulate copper homeostasis	[97,98,99]

### 3.1. The Unfolded Protein Response (UPR)

ER is the site for post-translational modifications and folding of secretory and membrane proteins. Alteration in Ca^2+^ homeostasis, inhibition of protein glycosylation, glucose deprivation or hypoxia can disturb normal ER function that results in an accumulation of unfolded/misfolded proteins. The adaptive response to ER stress, referred to as the unfolded protein response (UPR), consists in an inhibition of protein translation, an increase in ER chaperone expression and the activation of misfolded protein degradation process [83]. In the case of important cellular damages, ER stress initiates the cell death program through the activation of caspase-2 or apoptotic mitochondria pathway [27,83]. ER stress induces an IRES-dependent activation of cIAP1 translation [4], as well as a transcriptional expression of cIAP1, cIAP2 and XIAP, which contribute to cell death protection [84,85]. Interestingly, ER stress can induce a proteolytic processing of the ER protein GSPT1/eRF3 (G1 to S phase transition protein/eukaryotic Release Factor 3) that releases an IBM motif recognized by IAPs. GSPT1/eRF3-cIAPs interaction selectively stimulates cIAP1 auto-ubiquitination and degradation, compromising the anti-apoptotic response [86]. This phenomenon could be partially behind the onset of cell death in the case of irreversible damages. 

### 3.2. Role of IAPs as Neuroprotective Molecules

Brain cells are very sensitive to hypoxia and ischemia injuries which could inflict irreversible damages. Moreover, they are huge oxygen consuming cells and *per se* privileged sites of production of reactive oxygen species (ROS) that have a direct impact on proteins and inevitably downstream effects on their functionality. Oxidative protein modifications (*i.e.*, carbonization, S-nitrosylation, nitration) take part in the progression and pathophysiology of neurodegenerative disorders [87]. IAPs may represent a neuroprotective mechanism to counteract the deleterious effect of cellular stress and to preserve cell integrity and functionality. Neuronal cell differentiation is accompanied by a decrease in APAF-1 expression and then a gain of control by XIAP [44]. Accordingly, deletion of XIAP sensitizes neuronal cells to apoptotic stimuli such as hypoxia [88] and XIAP-deficient mice have an exacerbated response to neonatal hypoxic-ischemic injury [89]. Interestingly, sex difference in the sensitivity to perinatal hypoxic-ischemic injury has been associated with the differential expression of XIAP [90]. Thus, delivery of XIAP-derived peptide has been proposed as an effective therapy of brain diseases such as cerebral ischemia [91,92,93]. Beside cell death protection, XIAP appears to regulate intracellular ROS (reactive oxygen species) level [94]. Neurons from XIAP-overexpressing transgenic mice as well as XIAP transfected neuronal PC6.3 cells show an up-regulation in mitochondrial antioxidative enzymes including superoxide dismutase 2 (SOD2) and thioredoxine 2 (Txn2), a decreased production of ROS and consequently a reduced oxidative stress after hypoxia-ischemia, irradiation or xanthine-xanthine oxidase treatment [94,95]. Studies performed in XIAP^−/−^ mouse embryonic fibroblasts (MEFs) demonstrated that the basal level of XIAP is critically involved in the regulation of intracellular ROS by controlling the level of expression of antioxidant enzymes [96]. Regulation of copper intracellular level, that is required for number of antioxidant enzymes activity, represents another piece of evidence of XIAP antioxidant protection [97,98,99]. Fibroblasts derived from XIAP-deficient mice contain reduced copper level [98]. XIAP appears to regulate copper homeostasis by promoting the ubiquitination of copper transporters such as COMMD1 (Copper Metabolism MURR1 (mouse U2af1-rs region 1) domain containing 1) [97,98] and CCS (copper chaperone superoxide dismutase 1) [99]. In turn, copper can directly bind XIAP and accelerates its degradation [100]. 

A neuroprotective effect of NAIP has been also demonstrated. NAIP1 deleted mice develop normally and do not exhibit consistent abnormalities. However, the survival of pyramidal neurons in the hippocampus is greatly reduced after kainic acid-induced limbic seizures [101]. Conversely, ectopic expression of NAIP attenuates the neuronal damage in an *in vivo* model of cerebral ischemia [102]. 

Taken together, these observations lend strength that XIAP and NAIP are crucial regulators of neuronal homeostasis by preventing cell death and cellular damages following brain injuries. 

### 3.3. Role of IAPs in DNA Damage Response (Figure 3)

Environmental or chemotherapeutic genotoxic stress triggers DNA strand breaks. They are very quickly detected by sensor molecules that initiate p53-dependent and -independent DNA damage response. This adaptive response consists in an arrest of cell proliferation in order to prevent the spread of damages, and the activation of DNA repair mechanisms. It is accompanied by the engagement of pro-survival pathways such as NF-κB, which counteracts cell death by inducing the expression of various anti-apoptotic genes. When DNA damages are irretrievable, the cell activates its cell death program. Low dose γ-irradiation or DNA damaging agents such as etoposide can induce a rapid IRES-dependent up-regulation of XIAP and cIAP1 [80,81,103]. A translational regulation of XIAP involves the physical interaction of MDM2 (murine double minute 2) and XIAP IRES [81]. MDM2 is an E3-Ub ligase, well known as a regulator of p53 stability. Cellular stress generated by irradiation trigger a dephosphorylation, then a cytoplasmic translocation of MDM2, promoting cell proliferation arrest. Once in the cytoplasm, MDM2 can directly bind the XIAP IRES and stimulates its activity [81]. cIAP1 and XIAP decrease the sensitivity of cells to radiation induced apoptosis. They appear to be important intermediates, connecting DNA damage to the canonical NF-κB activating pathway [71]. DNA double-strand breaks recruit and activate the serine/threonine kinase ATM (Ataxia telangiectasia mutated) that initiates cell cycle arrest and DNA repair signaling pathways. ATM is translocated from the nucleus to the cytoplasm where it interacts with TRAF6 and favors its Ubc13-mediated K63 ubiquitination. These Ub chains serve as a signal for the recruitment of (1) cIAP1 through its UBA domain, (2) TAB2/TAK1 complex and (3) IKK complex. This Ub platform allows post-translational modifications of IKKs including cIAP1-mediated ubiquitination of IKKγ, required for NF-κB activation [71]. XIAP is important for TAK1 activation and association with the IKK complex [66,70] allowing the phosphorylation of IKKβ by TAK1. An auto-ubiquitination and a proteasomal degradation of cIAP1 and XIAP by DNA damage have also been reported [14,25], giving rise to Ripoptosome formation and cell death [25]. This could account for cell survival escapement in case of irreversible damages, in order to ensure tissue integrity.

**Figure 3:**
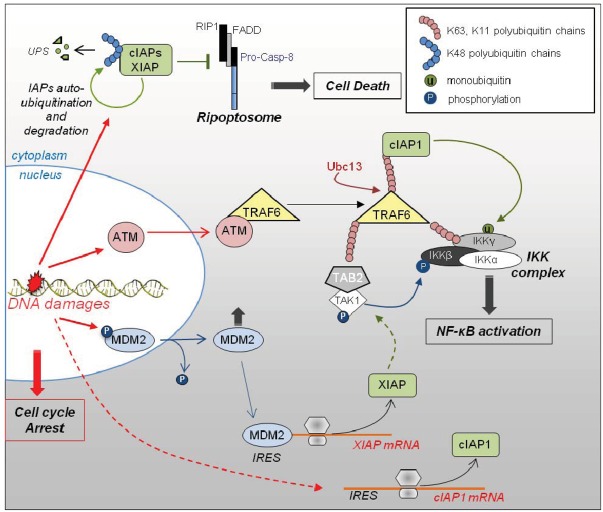
Role of IAPs in DNA damage response. Genotoxic stress-induced DNA strand breaks are very quickly detected by sensor molecules that activate cell cycle checkpoints and DNA repair mechanisms. They also induce IRES-dependent up-regulation of XIAP and cIAP1. MDM2 is dephosphorylated and exported from the nucleus to the cytoplasm where it physically interacts and stimulates XIAP IRES. cIAP1 and XIAP are regulators of DNA damage-mediated NF-κB activating pathway. The sensor protein ATM is translocated from the nucleus to the cytoplasm where it interacts with TRAF6 and favors its Ubc13-mediated K63 ubiquitination. These Ub chains are a signal for the recruitment of cIAP1, TAB2/TAK1 complex and IKK complex. TAK1 induces the phosphylation of IKKβ, cIAP1 mediates IKKγ mono-ubiquitination while LUBAC induces its linear ubiquitination. All of these post-translational modifications are required for NF-κB activation. XIAP controls this process by regulating TAK1 activation and association with the IKK complex. An auto-ubiquitination and degradation of cIAPs and XIAP have also been described, allowing the assembly of Ripoptosome leading to caspase-dependent or independent cell death. UPS: ubiquitin-proteasome system.

### 3.4. Role of IAPs in Adaptive Response of Cells to Pro-Inflammatory Environment

Cells of the monocytic lineage are central effectors of innate immune response. They are responsible for recognition and clearance of pathogens or infected cells and produce mediators of subsequent immune response including inflammatory cytokines, acid hydrolases and reactive oxygen or nitrogen species. They are very quickly recruited to the site of infection and represent the first line of defense against pathogens. They are therefore exposed to stressful environments containing exogenous pathogen-derived compounds, but also self-produced anti-microbial molecules which can act in an autocrine/paracrine manner. They are differentiated from common bone marrow progenitors into blood circulating monocytes and macrophages, which are subsequently activated by microbial compounds or cytokines [104]. The macrophage differentiation and activation programs are associated with the acquisition of resistance against cell death triggers as a result of an increase in the expression of anti-apoptotic proteins, a decrease in pro-apoptotic effectors and/or activation of survival pathways. An up-regulation of XIAP and cIAP1 expression have been observed along macrophage differentiation from bone marrow cells or monocytic cell lines [105,106,107,108] while an increase in cIAP2 has been reported after activation of macrophages and dendritic cells by LPS (lipopolysaccharides, component of gram-negative bacteria membrane) [109,110,111]. Moreover, differentiation signals induce a translocation of cIAP1 from the nucleus to the cytoplasm, which is correlated with a modification of its activity [76,77,112]. The function of cytoplasmic IAPs in inflammatory response as regulators of NF-κB-induced cytokine production is well described (reviewed in [2,3]). IAPs also appear to be important survival factors of macrophages in pro-inflammatory environment. Indeed, peritoneal and splenic macrophages from cIAP2^−/−^ mice lost their capacity to resist to apoptotic stimuli found in extracellular media after endotoxic injury that results to a decrease in the number of responsive macrophages [109]. Down-regulation of cIAPs considerably sensitizes bone marrow derived macrophages to HIV viral protein (Vpr) found in the serum of HIV-infected patients [113]. As already mentioned, IAPs also determine cell fate in response to TNFα pro-inflammatory cytokine or pathogen recognition by TLR, by favoring the activation of the NF-κB activating signaling pathway and inhibiting the assembly of cell death activating platforms [2].

## 4. IAPs and Disease

The adaptive response of cells to cellular stress, of endogenous or exogenous origin, is essential for maintaining cellular and tissue integrity and functionality. A deregulation of control mechanisms of homeostasis can favor the survival and growth of damage cells and promote tumorigenesis. Conversely, increased death response contributes to the development of degenerative diseases. Because of a central role of IAPs in cell fate along cellular stress, they are possibly involved in many diseases linked to a deregulation of cell homeostasis. Accordingly, IAPs have been involved in pathophysiology of cancer and neurodegenerative disorders. Moreover, cIAPs and XIAP have been involved in inflammatory diseases, because of their role in the regulation of TNFα signaling pathways and RIP1 activity and stability (for review, see [2,3]).

Overexpression of IAPs has been detected in number of tumor samples and correlated with bad prognosis or poor response to chemotherapy [20]. Oncogenic properties of cIAPs were demonstrated in mouse hepatocarcinoma [114], osteosarcoma [115], and mammary carcinoma [116] which all carry recurrent amplification at the mouse chromosome 9qA1 containing *cIAP1 (birc2)*, *cIAP2 (birc3)* and *Yap1 genes*. Such chromosome amplification (*amplicon 11q22*) also exists in human hepatocarcinoma [114], cervical [117], lung [118], oral squamous cell [119], and esophageal [120] carcinomas. The mechanisms of tumor promoting activity of IAPs are not completely solved and probably depend on a cellular model and stressful stimuli and may likely involve combined activities of IAPs. In mouse hepatocarcinoma, cIAPs seem to cooperate with c-myc to promote tumorigenicity [114]. In mouse breast cancer, a lack of Rb compensates genomic amplification of cIAPs [116]. Since both overexpression of cIAP1 [75] and down-regulation of Rb lead to a stimulation of E2F1, we may hypothesize that the capacity of cIAP1 to stimulate E2F1 transcription factor [75] is important for its oncogenic activity. The capacity of IAPs to regulate NF-κB survival and pro-inflammatory pathways is also an important factor that can contribute to tumor growth and metastasis. Mucosa-associated lymphoid tissue (MALT) lymphoma are typically associated with a chronic NF-κB activation and inflammation. Some (30%) MALT lymphoma are associated with a chromosomal translocation t(11;18)(q21;q21) generating a chimeric protein composed of N-terminal sequences of cIAP2 fused to C-terminal sequences of MALT1 and leading to uncontrolled NF-κB activity [121,122]. Other partners and ubiquitination targets of IAPs can also take part in tumor development and metastasis such as, for example, the c-RAF kinase or actin cytoskeleton regulators from the small RhoGTPase family [123,124].

Conversely, IAP inactivations were also associated with tumor development. *Birc2* or *birc3* genes, encoding cIAP1 and cIAP2, respectively, were mutated in some multiple myeloma sample [125,126] and *birc4* encoding XIAP in X-linked lymphoproliferative disease (XLP1) [127]. Multiple myeloma is a B cell malignancy characterized by a chronic NF-κB activation. In these cells, an inactivation of cIAPs induces NIK stabilization resulting in constitutive activation of the noncanonical NF-κB pathway involved in the pathophysiology of multiple myeloma [125,126]. 

Neurodegenerative disorders such as Alzheimer’s, Parkinson’s, Huntington’s disease or amyotrophic lateral sclerosis (ALS) are associated with excessive production and accumulation of ROS which inflict modification in intracellular proteins leading to misfolding, loss of functionality and neurotoxicity [87]. XIAP is a direct target of S-nitrosylation that affects its capacity to regulate caspases. S-nitrosylated XIAP accumulates in the brain of patients suffering Alzheimer’s, Parkinson’s and Huntington’s diseases [87,128]. In addition, a reduction in cytoplasmic XIAP and cIAPs has been found in Huntington’s disease brain tissue [129]. Both S-nitrosylation and reduced expression can contribute to neurodegeneration. Spinal muscular atrophy (SMA) is a neurodegenerative disorder characterized by a dysregulation of apoptosis in motor neurons. The NAIP gene is deleted in a significant proportion of patients suffering from SMA [19], and NAIP deletion appears to be associated with SMA severity [130].

Some alterations in XIAP, including reduced protein expression or the presence of gene mutations, have been detected in patients suffering from Wilson’s disease (WD) in which copper accumulation induces liver and brain injuries [100,131]. Since XIAP has been involved in copper metabolism [97,98,99], it may represent a pathogenic factor of WD.

## 5. Conclusion

Although IAPs were initially characterized as inhibitors of apoptosis through their ability to inhibit caspases activity, the physiological significance of this activity in mammals has long been discussed. cIAPs, XIAP and NAIP knockout mice are viable without any obvious abnormalities, however, the analysis of knockout mice derived cells revealed the importance of IAP in adaptive response to cellular stress. IAPs appeared to be able to convert the survival signal into a cell death inducing signal. Finally, recent findings have demonstrated a potent role of IAP in inflammation and innate immune response. Moreover, the increasing number of partners of IAPs identified suggests a very large spectrum of activity. Altogether, IAPs appeared to be important determinants of the response of cells to endogenous and exogenous cellular injuries, making the decision between cell adaptation or death. Consistent with these functions in preserving cellular and tissue homeostasis, deregulations in IAP expression were observed in tumors as well as in degenerative and inflammatory diseases. Mechanisms regulating IAP expression and activity are still poorly understood and more investigations are required to understand their real contribution in adaptive response of cells. Post-translational regulations such as phosphorylation, S-nitrosylation, ubiquitination, sumoylation, oligomerization, and subcellular localization, are likely very important for their activity and are poorly documented at the moment. IAP targeted therapy is a promising strategy for cancer treatment and is currently under clinical investigation [20]. However, given the wide spectrum of IAP activity, such therapy may have important consequences for immune system or central nervous system functions and will require more investigations in order to limit possible adverse impacts.
